# A comparison of behavioral and psychological characteristics of patients opting for surgical and conservative treatment for morbid obesity

**DOI:** 10.1186/s40608-016-0084-6

**Published:** 2016-02-05

**Authors:** Ingela Lundin Kvalem, Irmelin Bergh, Tilmann von Soest, Jan H. Rosenvinge, Tina Avantis Johnsen, Egil W. Martinsen, Tom Mala, Jon A. Kristinsson

**Affiliations:** 1 Department of Psychology, University of Oslo, PB 1094, Blindern, N-0317 Oslo, Norway; 2 Department of Psychology, University of Tromsø, Tromsø, Norway; 3 Center for Morbid Obesity and Bariatric Surgery, Oslo University Hospital, Postboks 4950, Nydalen, 0424 Oslo, Norway; 4 Department for Mental Health and Addiction, Oslo University Hospital, Postboks 4950, Nydalen, 0424 Oslo, Norway; 5 Institute of Clinical Medicine, University of Oslo, Oslo, Norway

**Keywords:** Psychosocial variables, Bariatric surgery, Weight treatment, Behavioral strategies

## Abstract

**Background:**

Little is known about the psychological prerequisites for weight loss maintenance after bariatric surgery. A first step in investigating whether existing knowledge of conservative weight loss treatment is applicable for lifestyle interventions postoperatively is to compare specific psychological characteristics at baseline. The aim of this study was to compare patients scheduled for bariatric surgery with patients receiving conservative treatment for morbid obesity on measures of behavioral and psychosocial characteristics considered predictors of their adoption of and adherence to long-term lifestyle recommendations.

**Methods:**

Baseline clinical and questionnaire data from the prospective “Oslo Bariatric Surgery Study” were used to examine potential differences between bariatric surgery patients (*n* = 301) and patients receiving conservative weight loss treatment (*n* = 261).

**Results:**

The surgical group was characterized by their younger age (43.8 vs. 46.2 years, *p* <0.01), higher percentage of women (79.1 vs. 70.1 %, *p* <0.05), and higher Body Mass Index (BMI; 45.0 vs. 41.9 kg/m^2^, *p* <0.001). A multiple logistic regression analysis, adjusting for group differences in BMI, gender, and age, showed that the surgical group had higher self-efficacy (Odds ratio; OR = 3.44, 95 % Confidence interval; CI 1.65, 7.14), more positive outcome expectations (OR = 1.53, 95 % CI 1.23, 1.89), and plans that were more explicit for changing their eating behaviors (OR = 1.80, 95 % CI 1.06, 1.93). The surgical patients were also less ready to change physical activity levels (OR = 0.59, 95 % CI 0.48, 0.73), had tried more types of unhealthy weight loss methods in the past (OR = 1.16, 95 % CI 1.01, 1.33), drank soda more frequently (OR = 1.24, 95 % CI 1.02, 1.50), had fewer binge eating episodes (OR = 0.38, 95 % CI 0.20, 0.71), and had more depressive symptoms (OR = 1.19, 95 % CI 1.09, 1.29).

**Conclusions:**

Patients opting for bariatric surgery had more positive expectations of the treatment outcomes and stronger beliefs in their ability to achieve these outcomes. Those starting conservative treatment had stronger beliefs in readiness to change physical activity levels. Future studies should explore the effect of interventions for bariatric surgery patients, promoting postoperative physical activity and stress realistic outcome expectations. The potential effects of incorporating this knowledge in intervention strategies remain to be explored.

## Background

The role of psychosocial factors in achieving sustained weight loss after bariatric surgery is unclear [1, 2]. In general, the individual’s ability to regulate behavioral changes, such as diet and physical activity, are considered factors central to achieving sustained weight loss after both surgical and non-surgical weight loss interventions [1, 3–6]. Yet, little is known about the psychological prerequisites for weight loss maintenance after bariatric surgery. Most studies investigating the psychological differences between patients treated surgically and non-surgically for obesity have focused on psychopathological factors, such as depression, anxiety, eating disorders [7–14], or problematic eating behaviors [7–10, 12]. The most consistent finding is that surgical patients more frequently report high levels of depressive symptoms than non-surgical patients [7, 10, 12, 14], although lower frequency [8], and no group differences [9, 11] also have been found. To our knowledge, only two comparison studies [10, 14] have included factors considered to be central for the adoption of and adherence to long-term lifestyle-behavior changes, such as self-efficacy, motivation, goal attainment, social support, and previous behavior [15, 16]. However, no firm conclusions about group differences can be drawn because the studies differed in the measures used, control variables included, e.g. Body Mass Index (BMI), and how the comparison samples were defined.

Literature reviews of bariatric surgery outcomes reveal varying results when individual psychological predictors for weight loss and weight loss maintenance are studied [17–19]. This inconsistency might be due to the preoperative psychological predictors that are most frequently studied, such as psychopathology, may influence weight loss mediated by the patients’ ability to adhere to postoperative behavior recommendations [1, 20]. Other explanations include small sample sizes, sample heterogeneity, and short follow-ups [1]. Due to the complex mechanisms of weight loss maintenance, it is necessary to include a wider range of psychological predictors in studies of postoperative behavior change [1, 5, 6], and incorporate short- versus long-term predictors of postoperative weight loss [20–22]. Sarwer et al. [3] argue that knowledge gained from conventional behavior-based weight interventions has often been dismissed too early in the long-term treatment of bariatric surgery patients.

An important component in reaching a long-term goal is strengthening the ability to exercise self-regulation, which typically is defined as the voluntary control of the initiation or inhibition of a behavior together with mental control by regulating thoughts, emotions and attention [23]. Factors that are related to self-regulation may thus also enhance behavior aimed at weight loss maintenance after bariatric surgery. Past behavior, self-monitoring, and self-efficacy are considered key components in self-regulation process [24]. Moreover, factors such as motivation for behavior change, weight loss goal, and expectations of weight loss outcome, have been related to adherence to recommended lifestyle changes after bariatric surgery [25].

Emotional and social distress is related to self-regulatory failure in chronic dieters. The explanation being that negative affect, such as depression, anxiety, body dissatisfaction, stress and social rejection, may decrease attentional control and thereby failure to control dieting [23]. While social support and family cohesiveness can promote self-regulation by providing a supporting environment for behavior change, it can also facilitate overeating [23].

A first step in investigating whether existing knowledge from conservative treatment is applicable for patients who have undergone surgical treatment is to compare specific baseline psychological characteristics that are relevant for predicting behavior changes in samples of surgical and non-surgical patients. Knowledge about valid and specific outcome predictors might facilitate individual treatment, follow-up, and the introduction of preventive measures.

The objective of this study was thus to compare baseline behavior and psychological characteristics, such as self-efficacy, motivation, goal attainment, mental health, and social support, in patients awaiting bariatric surgery and non-surgical weight loss treatment.

## Methods

### Participants

The data are retrieved from the “Oslo Bariatric Surgery Study” (OBSS), which is a prospective study of two cohorts of patients that will be followed over a 10-year period. The OBSS focuses on identification of psychosocial predictors of behavioral change and weight loss maintenance. All participants are assessed with self-report questionnaires at five time points; pretreatment (baseline), 1, 3, 5, and 10-years post-treatment.

**Fig. 1:**
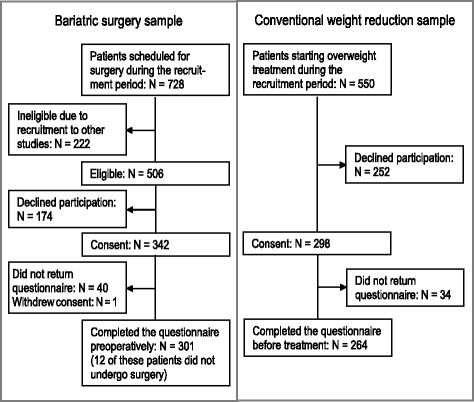
Flow chart of the recruitment process

Two groups of patients were recruited: patients scheduled for bariatric surgery (surgical group) and patients starting a conservative weight reduction treatment (non-surgical group). The inclusion criteria for both groups were BMI ≥40 or BMI ≥35 kg/m^2^, with obesity related comorbidity, age ≥18 years, and the ability to understand and comply with the study procedures. The patients in the surgery group had previous failed attempts of sustained weight loss using conservative measures. Only patients with current, previous established or suspected psychiatric disorder were evaluated by a psychologist or a psychiatrist. There was no routine preoperative psychological screening. The patients were recruited from the Center for Morbid Obesity and Bariatric Surgery at Oslo University Hospital, between February 2011 and September 2013, after they had participated in a preoperative mandatory course. The course consisted of 36 h (10 meetings), which included topics like treatment options, diet, physical activity, emotions, motivation, and behavior change. Of the total number of patients awaiting bariatric surgery during the study period (*N* = 728, 79.4 % women, mean age = 46.4, SD = 9.6, and mean BMI = 45.8, SD = 6.4), 222 were excluded due to their inclusion in other ongoing studies (see Fig. 1). Of the remaining 506 patients, 318 consented to participate, of whom 301 (49.5 %) responded to the pre-surgery questionnaires. Of the intention to treat sample used in this study, 12 respondents (4 %) did not proceed with surgery but were not excluded as they did not differ on any of the study variables.

Patients in the non-surgical group were recruited between February 2011 and June 2014 from two rehabilitation centers: the Tonsåsen Rehabilitation Center and the Haugland Rehabilitation Center. At both centers, the treatment programs combine diet, physical activity, and counseling, during three 2-week residential stays over an 8-month period. Of the 420 patients at the Tonsåsen Rehabilitation Center, and the 130 patients at the Haugland Rehabilitation Center, 207 (48.6 %) and 57 (43.8 %) respectively, agreed to participate in the present study and completed the questionnaires prior to the start of treatment. Data from both samples were collapsed as the patients from both rehabilitation centers did not differ according to gender, age, education, or BMI (*p* > 0.05). The total response rate was 47.4 % (*N* = 261). 

The Regional Ethics Committee for Medical Research (2009/1248a) approved the study protocol and all participants gave informed written consent before enrollment.

### Assessments

*Socio-demographic variables* consisted of gender, age, educational background, and partner status. Weight was measured using a platform scale (Seca 635, III; 0–300 kg), with patients wearing light clothing and no shoes, on the day of approval for bariatric surgery and the first day of conservative treatment. Height was measured using a wall-mounted adjustable instrument.

Two *family obesity indices* consisted of two questions about participants’ childhood and family members (parents/siblings) with obesity, and two questions about their partners and offspring with obesity, with the response options of (0) no and (1) yes. The sum of the response of the two questions in each index ranged from 0 (none with obesity), to 2 (both with obesity).

#### Current behavior and dieting history

*Current unhealthy eating habits* were assessed using four items specifically constructed for this study (frequency of snacking between meals, snacking on sweets between meals, drinking soda between meals, and night eating) with response options ranging from (1) never to (5) always. *Physical activity* during the previous week was assessed using the International Physical Activity Questionnaire (IPAQ)-Short Form [26]. The mean scores were calculated by weighting the type of activity by energy requirements and reported as metabolic equivalent values (MET). *Alcohol consumption* during the past 12 months was assessed by measuring the frequency of consuming one or more units of alcohol during the past year, with response options ranging from (1) never consumed alcohol to (9) daily or almost daily consumption. Intoxication was evaluated using response options ranging from (0) no alcohol use to (5) visibly intoxicated 10 times or more. *Self-monitoring of weight* was measured by one item with response options ranging from (1) almost never to (7) more than once a day. *Dieting and weight loss history* were measured by single questions from the Survey for Eating Disorders (SED) [27] and Weight and Lifestyle Inventory (WALI) [28]. The questions addressed the number of past successful weight loss attempts >10 kg, the total number of times patients had participated in organized weight loss programs, and the number of times during the past year patients had been on a self-initiated diet lasting for 3 days or more. We also calculated the percentage of years of dieting relative to age. *Weight loss methods* were assessed using 11 items with the response options of (0) no and (1) yes [27]. Based on exploratory factor analyses, three types of methods were identified i.e., *restricting the amount of food, restricting the type of food* (e.g., fat or sugar), and *unhealthy strategies* (e.g., using laxatives, drugs, or vomiting).

#### Motivation, goal attainment, and expectations

*Motivation to lose weight* was measured with the response to a single question, ranging from (1) not to (10) extremely motivated; patients were also requested to rate the degree of *social influence* on their decisions to seek treatment, on a scale from (1) no influence to (5) strong influence. *Readiness to restrict food intake* and *readiness to increase physical activity* were measured on a scale from (1) not ready to (10) trying to change, extrapolated from the Readiness and Motivation Interview [29] and the trans-theoretical model of change [30]. *Weight loss goal* was measured using one question from the Goals and Relative Weight Questionnaire [31], from which the relative difference (%) between participants’ actual weight before treatment and their goal weight was calculated. A higher percentage indicates a higher expectation of weight loss. *Outcome expectations* were operationalized by asking the respondents to “indicate how likely you believe it is that you will feel this way three years after the operation/treatment” on a set of 9 items with scores ranging from (1) no to (10) high expectations. The scale was developed for this study. The factor analysis of the responses yielded two factors: *well-being* (e.g., satisfied with the amount of weight lost, general appearance, self-esteem, and feeling good about oneself), and *social competence* (e.g., improved sex life, being outgoing, personal success, and fewer concerns). *General perceived self-efficacy*, i.e., a strong belief in one’s ability to master new behaviors or situations, was assessed using the 10-item General Perceived Self-efficacy Scale [32].

#### Self-evaluation, emotional distress, protective factors, and social environment

*Body image* was evaluated using two subscales of the Multidimensional Body-Self Relations Questionnaire: the Body Areas Satisfaction Scale (*BASS*; 9 items) and Appearance Orientation (14 items) [33]. A high mean score on the *BASS* indicates satisfaction with body and weight, while a high mean score on the subscale *Appearance Orientation* signifies preoccupation with one’s appearance and body. *Self-esteem* was assessed using a 4-item short version of Rosenberg’s Self-Esteem Scale [34]. The Emotion Regulation Questionnaire [35] was used to measure the ability to *regulate negative emotions* by redefining the situation (6 items) or by merely suppressing them (4 items). *Anxiety and depression symptoms* were measured using the Hospital Anxiety and Depression Scale (HADS) [36]. Higher sum score (0–21) reflects higher level of anxiety and depression symptoms. A cut-off score >10 indicates a probable anxiety or mood disorder. The frequency of *binge eating* episodes in the past 3 months was assessed using one item from the Survey for Eating Disorders [37], scored as (1) never, (2) previously, or (3) now, in conformance with the DSM-IV (Diagnostic and Statistics Manual for mental disorders) definition of binge eating symptoms. *Stressful life events*, *impact*, and *coping* were assessed with a scale developed at the University of Tromsø by measuring the frequency of having experienced 17 negative events (e.g., death or illness in the family, job loss, divorce, violence, and sexual abuse). The impact of each event was rated by participants from (1) very low to (4) very high, and coping with the event was rated from (1) badly to (3) well. *Relationship satisfaction* was measured using the 5-item Relationship Assessment Scale [38] with scores ranging from (1) little to (4) much satisfaction. The Resilience Scale for Adults [39] was used to assess factors that may protect against maladjustment. It consists of the five subscales: *family cohesion* (6 items), *social competence* (7 items), *social resources* (7 items), *personal strength* (10 items), and *structured style* (4 items). All items had a semantic differential scale format with scores ranging from 1 to 7.

### Statistical analyses

Analysis of covariance (ANCOVA) was used to compare the differences between the two study groups on all of the variables. As there were statistically differences in gender, age, and BMI between the two study groups, the group means were adjusted for possible confounders by entering gender, age, and BMI as covariates. The effect size, partial eta squared (ηp^2^) was reported, indicating the proportion of variance explained by a variable that is not explained by the covariates. The magnitude of the effect size for the partial eta squared is 0.01 (small), 0.06 (medium), and 0.14 (large), according to Cohen’s guidelines [40]. Multiple logistic regression analyses were conducted to identify the variables that uniquely predicted group membership. In these analyses, all study variables showing significant mean differences between the two treatment groups (*p* < 0.05) in the ANCOVA were included simultaneously, while controlling for BMI, age, and gender.

## Results

Compared to the non-surgical group, patients in the surgical group were more frequently women (*p* < 0.05), were younger (*p* < 0.01), and had a higher mean weight and BMI (*p* < 0.001) (Table 1).

**Table 1:** Descriptive statistics of patients opting for non-surgical and surgical obesity treatment (*N* = 562)

		Non-surgical	Bariatric surgery	
		*n* (%)	*n* (%)	*P*
Number of subjects		261	301	
Women		183 (70.1)	238 (79.1)	<0.05
Men		78 (29.9)	63 (20.4)	
Have a partner/ married		166 (64.6)	193 (65.7)	0.80
Education				
< 10 years		45 (17.4)	64 (21.7)	
10–12 years		118 (45.7)	145 (49.2)	
> 12 years		95 (36.8)	86 (29.2)	0.13
	Range	Mean (SD)	Mean (SD)	
Age (years)	20–73	46.2 (10.8)	43.8 (9.6)	<0.01
Weight (kg)	86–222	123.6 (1.2)	130.3 (1.1)	<0.001
BMI (kg/m^2^)	31–70	41.9 (5.4)	45.0 (6.0)	<0.001

Note: Group differences tested by Chi-square and independent-samples *t*-test

Patients belonging to the surgical group reported a higher frequency of drinking soda (*p* < 0.01), more use of unhealthy weight-reduction methods (*p* < 0.001), and a family history of obesity (*p* < 0.01). In addition, they had a longer history of dieting (*p* < 0.001), participated more often in organized weight loss programs (*p* < 0.01), and had more often lost >10 kg (*p* < 0.01) (Table 2).

**Table 2:** Behavior and dieting history between patients opting for non-surgical and surgical obesity treatment

		Non-surgical *n* = 261	Bariatric surgery *n* = 301	Adjusted for gender/age/BMI		
	Range	Mean (SD)	Mean (SD)	F	*P*	ηp^2^
Family obesity						
- parent/sibling + as a child	0–2	1.0 (0.7)	1.3 (0.7)	8.98	<0.01	0.017
- partner + own children	0–2	0.7 (0.7)	0.7 (0.6)	0.01	0.93	
Current behavior						
Unhealthy eating habits						
- Total	1–5	2.4 (0.7)	2.5 (0.7)	0.73	0.39	
- Snacking between meals	1–5	3.0 (0.9)	3.1 (1.0)	0.82	0.37	
- Snacking on sweets	1–5	2.8 (1.0)	2.8 (1.0)	1.13	0.29	
- Drinking soda drinks	1–5	2.6 (1.4)	3.0 (1.4)	6.71	<0.01	0.012
- Night time eating	1–5	1.3 (0.7)	1.2 (0.6)	3.57	0.11	
Physical activity MET-minutes/week	0–14,958	2292.5 (2445.3)	1844.2 (2290.6)	3.96	<0.05	0.008
Alcohol use	1–9	4.5 (1.8)	4.0 (1.7)	2.12	0.14	
Self-monitoring of weight	1–7	3.1 (1.5)	2.7 (1.5)	3.70	0.06	
Dieting history						
% yrs. Dieting	0–88	47.6 (20.1)	53.9 (17.4)	11.93	<0.001	0.023
# organized diet programs	0–25	2.7 (2.9)	3.6 (4.1)	7.31	<0.01	0.014
# 3 day diets past year	0–25	2.5 (4.2)	3.2 (4.6)	1.07	0.30	
# lost >10 kg	0–12	3.1 (2.8)	4.2 (3.4)	8.36	<0.01	0.017
Diet strategies						
-Total	0–11	4.0 (2.3)	4.9 (2.3)	13.75	<0.001	0.025
- Less fat/carbs/sweets	0–3	1.6 (1.1)	1.8 (1.0)	1.28	0.26	
- Skipping meals/eat less/fasted	0–3	1.3 (0.9)	1.5 (0.9)	6.89	<0.01	0.013
- Use laxatives/ vomiting/diet drugs/diuretic drugs	0–4	0.6 (0.8)	1.0 (0.9)	16.75	<0.001	0.030

Note: # number, *SD* standard deviation, Estimate of effect size: ηp^2^ = Partial Eta-squared

The surgical group reported higher general self-efficacy (*p* < 0.001), reflecting a stronger belief that their actions would result in a successful outcome, whereas the non-surgical patients indicated they more frequently were influenced by others in their decision to seek treatment (*p* < 0.001). The surgical patients had more specific plans for changing their eating behaviors, as well as plans for coping with barriers and setbacks (*p* < 0.001). Moreover, they expected their well-being and social competence to improve to a greater degree in the next three years. Surgical patients had a relatively higher weight loss goal, but they were to a lesser degree ready to increase their physical activity, compared to the non-surgical group (*p* < 0.001). Most of the significant mean group differences had small to medium effect sizes. Notably, no group differences were detected in motivation for losing weight (Table 3).

**Table 3:** Motivation, goal attainment, and expectations between patients opting for non-surgical and surgical obesity treatment

			Non-surgical *n* = 261	Bariatric surgery *n* = 301	Adjusted for gender/age/BMI		
	Range	α	Mean (SD)	Mean (SD)	F	*p*	ηp^2^
Motivation for weight loss	1–10		9.1 (1.1)	9.2 (1.4)	0.03	0.87	
Social influence on treatment decision	1–5		2.6 (1.1)	2.3 (1.2)	12.22	<0.001	0.023
Readiness for change							
- eat less	0–10		9.0 (1.2)	9.0 (1.3)	0.03	0.86	
- less overeating	0–10		8.2 (2.6)	8.4 (2.5)	0.43	0.51	
- increase activity	0–10		9.0 (1.2)	8.4 (1.6)	26.11	<0.001	0.047
General self-efficacy	1–4	0.88	2.9 (0.5)	3.1 (0.4)	20.23	<0.001	0.037
Action + Coping planning							
- eating	1–4	0.86	2.8 (0.5)	3.0 (0.5)	23.92	<0.001	0.043
Action + Coping planning							
- physical activity	1–4	0.92	2.6 (0.6)	2.8 (0.6)	2.68	0.10	
Weight loss goal							
- % happy weight	2–60		29.8 (9.4)	37.9 (8.0)	59.76	<0.001	0.101
Future expectations							
- well-being	1–10	0.88	7.6 (1.8)	8.4 (1.4)	38.08	<0.001	0.067
- social competence	1–10	0.71	6.8 (1.9)	7.2 (1.8)	8.74	<0.01	0.016

Note: *α* Cronbach’s alpha, *SD* standard deviation, Estimate of effect size: ηp^2^ = Partial Eta-squared

Overall, there were few significant group differences regarding self-evaluative and mental health factors (Table 4). The surgical patients reported a higher level of depressive symptoms (*p* < 0.05) and fewer binge-eating episodes (*p* < 0.01). Significantly more patients (11 %) in the bariatric surgery group than in the non-surgical group (4 %), exceeded the cut-off level of HADS (>10) for a probable depressive disorder (*χ* ^2^ = 10.34, *p* < 0.001). The number of participants above the cut-off score on the anxiety subscale was high in both groups (bariatric surgery = 21 %; conservative weight loss treatment = 22 %).

**Table 4:** Self-evaluation, emotional distress, and protective factors in patients opting for non-surgical and surgical obesity treatment

			Non-surgical *n* = 261	Bariatric surgery *n* = 301	Adjusted for gender/ age/BMI		
	Range	α	Mean (SD)	Mean (SD)	F	*p*	ηp^2^
Self-esteem	1–4	0.81	2.7 (0.7)	2.7 (0.7)	0.54	0.46	
Body satisfaction	1–5	0.74	2.4 (0.5)	2.6 (0.5)	1.76	0.18	
Appearance importance	1–5	0.82	3.4 (0.7)	3.4 (0.8)	2.68	0.10	
Mental health							
- Anxiety	0–20		7.2 (4.1)	7.0 (4.2)	0.78	0.38	
- Depression	0–20		4.8 (3.2)	5.4 (3.8)	4.45	<0.05	0.008
Binge eating	1–3		1.8 (0.9)	1.7 (0.8)	7.69	<0.01	0.015
Stressful life events							
-frequency	0–13		3.3 (2.2)	3.8 (2.8)	3.25	0.07	
-impact	1–4		3.2 (0.7)	3.3 (0.7)	0.59	0.44	
-coping	1–3		1.9 (0.6)	2.0 (0.6)	1.41	0.24	
Emotion regulation							
- reappraisal	1–7	0.77	4.1 (0.9)	4.0 (0.9)	1.48	0.22	
- suppression	1–7	79	3.8 (1.1)	3.8 (1.1)	0.34	0.56	
Resilience factors							
- Total	1–5	0.91	3.6 (0.6)	3.7 (0.6)	3.00	0.08	
- Personal strength	1–5	0.87	3.4 (0.8)	3.5 (0.8)	8.40	<0.01	0.015
- Structural style	1–5	0.49	3.2 (0.8)	3.4 (0.8)	10.45	<0.001	0.019
- Social competence	1–5	0.74	3.6 (0.7)	3.7 (0.8)	0.99	0.32	
- Family cohesion	1–5	0.84	3.7 (0.8)	3.6 (1.0)	0.18	0.67	
- Social resources	1–5	0.80	4.1 (0.7)	4.1 (0.8)	0.01	0.95	
Relationship satisfaction^a^	1–7	0.85	5.6 (1.1)	5.6 (1.1)	0.07	0.79	

Note: *α* Cronbach’s alpha, *SD* standard deviation; Estimate of effect size: ηp^2^ = Partial Eta-squared. ^a^Relationship Satisfaction Scale was only completed by respondents who were in a relationship (*n* = 190/165)

The surgical group exhibited more dispositional resilience, i.e., personal competence in terms of self-esteem, hope, determination, and a realistic life orientation (*p* < 0.01) and structured style (the ability to keep daily routines, to plan, and to organize) (*p* < 0.001), compared to the non-surgical group (Table 4).

Multiple logistic regression analyses indicated that the probability of belonging to the surgical group was uniquely related to drinking soda more frequently, using more unhealthy weight loss methods, having more depressive symptoms, a lower likelihood of recent binge-eating episodes, and less readiness to increase physical activity. On the other hand, being in the surgical group also was related to several motivational psychological factors, such as higher self-efficacy, having specific plans to cope with the challenges of changing their eating behavior, having a higher weight loss goal, and expecting improved well-being in the future (Table 5).

**Table 5:** Multiple logistic regression analysis for variables predicting the likelihood of belonging in the bariatric surgery group (1) (vs. non-surgical treatment group = 0)

	*n* = 430		
Variable	OR	95 % CI	*p*
Gender (Men = 0; Women = 1)	*1.48*	*[0.74, 2.97]*	*0.26*
BMI	*1.00*	*[0.93, 1.08]*	*0.98*
Age	*0.98*	*[0.95, 1.01]*	*0.13*
Family obesity			
- obese parent/sibling *and* as a child (1) vs. none (0)	1.99	[0.92, 4.31]	0.08
- obese parent/sibling *or* as a child (1) vs. none (0)	1.36	[0.67, 2.72]	0.39
Unhealthy eating habits: frequency soda drinks	1.24	[1.02, 1.50]	<0.05
% years dieting	1.01	[0.99, 1.03]	0.39
# organized diet programs	1.05	[0.96, 1.15]	0.28
# times lost >10 kg	1.02	[0.93, 1.12]	0.68
Total number of diet strategies used	1.16	[1.01, 1.33]	<0.05
Social influence on treatment decision	0.83	[0.65, 1.05]	0.12
Readiness for change-increase physical activity	0.59	[0.48, 0.73]	<0.001
General self-efficacy	3.44	[1.65, 7.14]	<0.001
Coping planning: eating	1.80	[1.06, 3.03]	<0.05
Weight loss goal: % happy weight loss	1.05	[1.02, 1.08]	<0.01
Future expectations			
- positive emotions	1.53	[1.23, 1.89]	<0.001
- improved social life	0.89	[0.75, 1.06]	0.20
Depressive mood	1.19	[1.09, 1.29]	<0.001
Binge eating			
- now (1) vs. never (0)	0.38	[0.20, 0.71]	<0.01
- previously (1) vs. never (0)	0.68	[0.34, 1.67]	0.29
Resilience factors: personal strength	1.02	[0.61, 1.70]	0.95
Resilience factors: structural style	1.38	[0.98, 1.96]	0.07

Note: Covariates in italics; *OR* Odds ratio, *CI* Confidence interval

## Discussion

The present study extends previous research by investigating differences in behavioral and psychological factors between obesity treatment groups, adjusted for BMI, gender, and age. Many of the baseline behavioral and psychological characteristics related to motivation, goal attainment, future life expectations, and depression, differed significantly between patients awaiting bariatric surgery and patients in a conservative weight loss programs. More specifically, patients in the surgical group had a history of longer, more frequent, and unsuccessful experiences with dieting by using unhealthy weight loss methods, particularly in terms of skipping meals or taking laxatives. These group differences probably reflect failed attempts using conservative weight loss methods. Chronic dieting has also been documented as a risk factor for more problems with lifestyle changes after surgery [41].

The most notable group differences were that patients opting for bariatric surgery scored higher on factors considered central to initiating and maintaining behavior change [15, 16], such as higher general self-efficacy, weight loss goals, and expectations of increased well-being in the future, as a result of the surgery. Perceived general self-efficacy is influenced by previous behavioral successes and failures, which may affect future behavioral change attempts [32]. Despite a history of failed weight loss attempts, the surgical patients had a stronger belief in overcoming future obstacles to behavioral change than non-surgical patients. High self-efficacy has been related to adherence with postsurgical lifestyle recommendations [42]. Although self-efficacy did not differ from that of the non-surgical patients in a study by Ahnis et al. [10], they did find more active coping (which includes making plans) in patients seeking bariatric surgery, which is in accordance with our results. Wadden et al. [14] also found no difference in self-efficacy, but higher motivation and weight loss goals in a female bariatric surgery group (obesity class III), compared to a group of non-surgical patients (obesity class I-II). However, the observed findings in their study may be attributable to the higher obesity level of the surgery group, as the data were not adjusted for the difference in BMI. Planning and goal attainment are factors that have been shown to strengthen the motivation and long-term capability for change in diet behavior and physical activity [43, 44]. To our knowledge, the present study is the first one to compare future life expectations between surgical and non-surgical patients. Favorable outcome expectations, which characterized our bariatric surgery group, are generally considered to be important for the initiation of behavior change [24].

Similar to the study procedures of Ahnis et al. [10], all patients approved for bariatric surgery in this study had completed a mandatory course before recruitment. During the course, factors such as expectations and planning how to change and cope with the post-surgical diet were discussed, which might have influenced their response patterns. Although increased physical activity was a topic included in the course, the patients in the bariatric surgery group were less ready to be physically active in the future than those in the non-surgical group. This finding might reflect a belief that bariatric surgery will enable weight loss without the need to change lifestyle [45]. Although the group difference in current physical activity was negligible in this study, in accordance with Rutledge et al. [8], readiness for behavior change is thought to be an essential first motivational step [30].

The prevalence of probable depression (9 %) and anxiety disorders (21 %), found in the bariatric surgery group is comparable to the baseline prevalence reported in the Swedish Obesity Subjects (SOS) study (4 and 21 % respectively) [7]. As in our study, the SOS study found higher mean levels of depressive symptoms in the bariatric surgery group compared to the non-surgical group, and no group difference in anxiety symptoms [7]. Negative affect can make it more difficult for some patients to simultaneously regulate their emotions and behavior or different behaviors (e.g., both diet and exercise), thereby disrupting their ability to control behavior change [22, 24]. In contrast to never having a binge episode, current binge eating was associated with a higher probability of belonging to the conservative weight loss treatment group. This finding contrasts other studies which report more binging among patients undergoing *bariatric surgery* [11, 12], no group differences in problem eating behaviors, such as disinhibition [7, 9], or negligible group differences in binge eating [13]. Such mixed findings might be due to the use of different assessment instruments. Moreover, some patients may underreport serious eating disorders to avoid being excluded from the surgical option [46], but the difference found in this study was small and should be interpreted with caution.

The pretreatment differences in psychological predictors indicate that the results of studies addressing effective behavior change and weight loss maintenance programs for conservative weight loss treatment patients may, with some adjustments, also apply to patients undergoing bariatric surgery [3]. Future interventions for patients opting for bariatric surgery may emphasize the importance of physical activity, in particular, for postoperative weight loss maintenance, and reinforcing patients’ motivation for long-term lifestyle changes also after surgery. Postoperative improvements in depressive symptoms are largely dependent on the degree of weight loss, which does not seem to be the case for anxiety symptoms [47, 48]. If weight loss is less than expected, or if patients put on weight, it will be especially important to prevent patients from returning to unhealthy weight loss methods. Our findings imply that future research may examine how high outcome goals and expectations interact with depressive symptoms depending on the degree of weight loss throughout the post-operative course.

A strength of our study is that, due to free health care in Norway, the choice of treatment reflects only medical evaluations and patient preferences, and not economical or insurance factors. The relatively large study samples are also representative of the two treatment cohorts of patients with morbid obesity. However, limitations of the study should be noted. Only about half of those invited to participate completed the questionnaire and it was not possible to assess whether respondents differed from non-respondents on psychological characteristics. However, as the response rates in the surgical and non-surgical groups were similar, group differences most probably cannot be explained be different response rates. Moreover, the interval between recruitment and treatment differed between the two groups. The surgical group completed the questionnaire up to several months before surgery, but after being approved for surgery and having attended the mandatory course; the non-surgery group, however, completed the questionnaire at the start of treatment at the rehabilitation center. Another limitation is that the large number of variables increases the probability of statistical Type I errors. Our findings should also be interpreted with caution because of the cross-sectional design and the use of data collection methods mainly based on self-report. Finally, although the majority of measures used were validated, some of the instruments were developed specifically for this study and their psychometric properties were unknown.

## Conclusion

In this comprehensive comparison study of baseline behavioral and psychological characteristics of patients scheduled for surgical and conservative weight loss treatment, the focus was specifically on factors central to the adoption of and adherence to long-term lifestyle behavior change. Patients opting for bariatric surgery had more positive expectations about the treatment outcomes and a stronger belief in their ability to achieve these outcomes. The patients starting conservative treatment had stronger beliefs in their readiness to change their physical activity levels. Future studies should explore interventions for bariatric surgery patients which incorporate the promoting of postoperative physical activity and which link outcome expectations to health behavior change.

### Availability of data and materials

The data will not be shared because the Data protection official at the Oslo University Hospital does not allow sharing the dataset.
